# No Evidence That Vitamin D Levels or Deficiency Are Associated with the Risk of Open-Angle Glaucoma in Individuals of European Ancestry: A Mendelian Randomisation Analysis

**DOI:** 10.3390/genes15081084

**Published:** 2024-08-16

**Authors:** Nour Kanso, Munisa Hashimi, Hasnat A. Amin, Alexander C. Day, Fotios Drenos

**Affiliations:** 1 Department of Life Sciences, College of Health, Medicine and Life Sciences, Brunel University London, Uxbridge UB8 3PH, UK; 1822392@alumni.brunel.ac.uk (N.K.); munisa.hashimi@brunel.ac.uk (M.H.);; 2 Moorfields Eye Hospital, London EC1V 2PD, UK; alex.day1@nhs.net; 3 UCL Institute of Ophthalmology, London EC1V 9EL, UK

**Keywords:** vitamin D, open-angle glaucoma, mendelian randomisation, genetic epidemiology

## Abstract

Background: Glaucoma is the second leading cause of blindness worldwide, with intraocular pressure as the only known modifiable risk factor. Vitamin D has been proposed to influence intraocular pressure and decrease retinal ganglion cell degeneration. Based on these findings, vitamin D has been suggested to prevent or reduce the severity of primary open-angle glaucoma (POAG), which is the most common form. Methods: We applied two-sample Mendelian randomisation (MR) analyses to data from the SUNLIGHT consortium and the UK Biobank to assess the causal effect of vitamin D levels and vitamin D deficiency on primary open-angle glaucoma (POAG). MR analysis, including sensitivity tests using other GWAS summary statistics from FinnGen, was also performed. We also investigated the association between single nucleotide polymorphisms (SNPs) on genes involved in vitamin D metabolic pathways and POAG. Results: We found no statistical evidence that vitamin D levels (OR = 1.146, 95% CI 0.873 to 1.504, *p* = 0.326) or vitamin D deficiency (OR = 0.980 (95% CI 0.928 to 1.036, *p* = 0.471) causally affect the risk of developing POAG. Sensitivity analyses, including the use of a more relaxed *p*-value threshold, and use of winter-measured samples only, replication in the FinnGen dataset, and exploration of specific genetic markers also showed no evidence of association between SNPs for genes involved in key steps of vitamin D metabolism and POAG. Conclusions: These results indicate that vitamin D may not be a significant factor in modifying POAG risk, challenging the hypothesis that vitamin D supplementation could be effective in reducing POAG risk. Further research should focus on identifying other potential risk factors for POAG prevention strategies.

## 1. Introduction

Glaucoma is a group of optic neuropathies that cause the degeneration and dysfunction of retinal neurones. As glaucoma progresses, the optic nerve—made up of retinal ganglion cells (RGCs), soma and their axons—gradually degenerate, resulting in visual impairment. This irreversible process is the second leading cause of blindness worldwide [1]. Globally, over 70 million individuals are affected by glaucoma. This is expected to increase to 111 million in the next two decades [2]. Glaucoma is divided into two main subtypes: primary open-angle glaucoma (POAG) and primary angle-closure glaucoma (PACG) [3]. POAG is the most common subtype of glaucoma [3].

Vitamin D is a collection of lipid-soluble prohormones comprising two physiological types: ergocalciferol and cholecalciferol [4]. Both forms of vitamin D—whether generated upon ultraviolet B radiation (UVB) exposure or consumed from the diet—are hydroxylated in the liver, primarily by the cytochrome P450 (CYP) 2R1 and CYP24A1, to make 25-hydroxyvitamin D (also known as calcifediol, 25(OH)D). Whilst CYP2R1 is the main vitamin D 25-hydroxylase, mitochondrial CYP24A1 performs residual hydroxylation [5]. 25(OH)D undergoes a second process of hydroxylation in the kidneys with CYP27B1 to form 1α,25-dihydroxyvitamin D (1,25(OH)2D or calcitriol), which is biologically active. The closest estimate of one’s vitamin D levels is to measure 25(OH)D serum levels [6]. Vitamin D deficiency is commonly defined as levels less than 25 nmol/L, and vitamin D insufficiency as levels between 25 nmol/L and 59 nmol/L. In European populations, deficiency occurs in just under 20% of individuals in Northern European countries and affects between 30 and 60% of Western, Eastern and Southern European populations [7]. Older adults are at an increased risk of vitamin D deficiency or vitamin D insufficiency due to the reduced skin production of vitamin D as well as age-related factors, including being more housebound, that result in limited sun exposure [8,9,10].

The association between vitamin D and POAG—while mostly poorly understood—is of particular interest as vitamin D is a cheap and relatively safe supplement when consumed at the recommended dose. Vitamin D appears to have a protective role against reactive oxygen species (ROS) production and oxidative damage [11,12,13]. Therefore, theories correlating vitamin D with POAG include the vitamin’s ability to act as an antioxidant and its role in suppressing genes involved in inflammation [14]. The administration of 1,25(OH)2D to human retinal pigment epithelium cells was shown to decrease the effects of oxidative stress in the eye, therefore suggesting a protective role in the eye [15].

Previous studies have attempted to assess the association of vitamin D levels with POAG. Several cross-sectional and case–control studies associated lower 25(OH)D levels with POAG [16,17,18,19,20]. However, a recent meta-analysis conducted by Li et al. found no relationship between glaucoma and vitamin D in three independent studies [21]. However, these studies could not establish if the relationship was causal and if supplementation with vitamin D could be used as part of prevention. Confounding factors can falsely exhibit an association between two variables when there is no real causal link present [22]. Reverse causation is also possible, and it has been suggested that those with glaucoma are more likely to stay indoors due to the challenges of visual impairment, decreasing their UVB exposure and, therefore, vitamin D synthesis and levels [20].

Mendelian randomisation (MR) is an epidemiological method that allows the evaluation of a risk factor’s causal effect on a phenotype or outcome. Specifically, genetic variants in the form of single nucleotide polymorphisms (SNPs) are treated as proxy measurements of the risk factor of interest [23]. The fundamental idea behind MR is that paternal and maternal genetic variation is randomly assigned at conception due to meiosis in relation to possible confounders of the association [24]. Thus, a genotype that predisposes the circulating level of a biomarker of concern, in this case, vitamin D, can be considered as a randomised treatment in a naturally occurring randomised controlled trial, with randomised controlled trials considered the gold standard to assess the causal effect of a medical intervention. The recent popularity of MR is due to the ability to use reported estimates of association of a genetic variant with the relevant exposure and tested outcome to calculate the causal effect of the former on the latter as the ratio of the two coefficients. When multiple genetic variants are used, the overall causal effect is the weighted average of the ratios for all variants.

In this study, using published data from well-powered genetic studies, we performed a two-sample MR analysis to determine if vitamin D levels are causally associated with POAG and if vitamin D deficiency is a modifiable risk factor for POAG. Because of the heterogeneous nature of glaucoma, we explored the main clinical subtype, POAG, for a better understanding of possible vitamin D effects on its development.

## 2. Methods

### 2.1. Vitamin D Levels: The SUNLIGHT Consortium

We extracted genetic instruments (proxies of the exposure) for vitamin D levels in the form of single nucleotide polymorphisms (SNPs) from the genome-wide association study (GWAS) available from the SUNLIGHT consortium. The SUNLIGHT consortium tested 79,366 individuals of European descent from 31 epidemiological studies across Europe, Canada, and the USA. Vitamin D levels were determined as 25(OH)D serum concentration [25]. This study expanded on the work of the initial SUNLIGHT consortium by Wang et al. [26] that identified four associated genetic loci (*CYP24A1*, *CYP2R1*, *GC* and *NADSYN1/DHCR7*) and found two additional loci: *SEC23A* and *AMDHD1* [25,26]. The analysis, described elsewhere [25], was adjusted for the month of sample collection, body mass index, age, gender, and principal components for genetic ancestry. The estimates from different studies were combined through a fixed-effects inverse-variance-weighted meta-analysis [25].

### 2.2. Vitamin D Deficiency: The UK Biobank

To estimate the causal relationship between vitamin D deficiency on glaucoma, we obtained genetic proxies of vitamin D deficiency from Amin & Drenos, who performed a GWA analysis of the UK Biobank (UKBB) [27]. The UKBB is a cohort study of >500,000 UK residents collected between 2006 and 2010. UKBB assessment centres found across England, Scotland, and Wales gathered data from participants (aged 40–69 years at recruitment) through questionnaires, cognitive and physical examinations and collected samples from both the blood and urine [28]. Those with <25 nmol/L of measured 25(OH)D levels were considered as cases, and individuals with ≥50 nmol/L of 25(OH)D levels as controls. Following quality controls (QC), as described elsewhere [27], the analysis included 35,076 cases and 140,898 controls, all of European ancestry. Deficiency status was tested against the imputed and genotyped SNPs available, adjusting the model for the first four principal components of the genome, the age at baseline, sex and the genotyping array used [27].

### 2.3. Primary Open-Angle Glaucoma

We obtained summary statistics for POAG from the study conducted by Gharahkhani et al. [29]. This study used data from 16,677 cases and 199,580 controls of European descent to identify 127 loci associated with open-angle glaucoma. In this case, POAG was predominately defined based on the ICD9/ICD10 criteria, although a small number of self-reported cases were also included [29].The summary statistics were obtained from https://www.ebi.ac.uk/gwas/publications/33627673 (accessed on 27 June 2024).

### 2.4. Statistical Analysis

We used the statistical software “R” version 4.0.3 to run statistical analyses [30]. We used the “TwoSampleMR” R package version 0.5.5 to perform the MR tests [31].

Using the SUNLIGHT consortium GWAS summary level data from https://drive.google.com/drive/folders/0BzYDtCo_doHJRFRKR0ltZHZWZjQ (accessed on 27 June 2024) (as provided by Jiang et al. [25]), we applied the standard GWAS *p*-value threshold of 5 × 10^−8^ to identify the genetic variants correlated with vitamin D levels. We then clumped the SNPs to identify independent genetic variants with an R^2^ > 0.001, thereby removing correlated variants that could bias the MR estimates by double counting areas of high linkage disequilibrium [32]. As a result, we used seven genome-wide significant SNPs for vitamin D levels that were matched to the POAG data. The list of instruments used, together with their effect sizes, can be found in Supplementary Table S1A. For vitamin D deficiency instruments, we used the list of SNPs and estimates provided by Amin & Drenos. The authors provided the independently associated SNPs with an R^2^ > 0.001 [27]. After matching the 17 reported SNPs with the POAG data, a total of 15 SNPs remained, which we used as instruments for vitamin D deficiency. The list of instruments used for vitamin D deficiency, together with their effect sizes, can be found in Supplementary Table S1B.

We obtained causal estimates through MR using the inverse-variance-weighted (IVW) method [33]. This method combines estimates from multiple SNPs, assuming that all the SNPs used are valid independent instruments satisfying three main assumptions. These stipulate that the SNPs are associated with the exposure of interest; they are not associated with any of the known confounders, and if they are associated with the outcome, this is only through their effect on the exposure and not through an independent pathway. The first assumption is satisfied using replicated reported associations from well-conducted GWASs. The second assumption can be tested by looking for any associations between the selected instruments and known confounders through phenoscanner (https://github.com/phenoscanner/phenoscanner, accessed 10 October 2023). The third assumption is commonly the result of pleiotropy. Pleiotropy is the ability of genetic variants to influence more than one biological pathway. Such effects may mean that SNPs could impact POAG through a different trait or route than vitamin D, known as horizontal pleiotropy [34]. To test for horizontal pleiotropy, we used the MR Egger’s regression method, which provided both a causal estimate (slope) and a test for directional pleiotropy (intercept). If the intercept was not significantly different from zero (*p* ≥ 0.05), the IVW estimate was considered as a reflection of the true causal effect [35]. If we observed statistical evidence of pleiotropy (*p*-value for MR-Egger intercept < 0.05), additional pleiotropy robust MR methods, including the use of the simple mode, weighted mode, and weighted median meta-analysis methods, were also used. These methods refer to different models that can be used in the meta-analysis of individual ratios as alternatives to the classical meta-analysis method of using the mean.

To visually summarise the results, we produced forest plots using the R package “forestplot” version 1.10.1 (https://cran.r-project.org/web/packages/forestplot/vignettes/forestplot.html, accessed on 27 June 2024). To see the causal estimates of the individual SNPs for each exposure on each outcome, we used the function mr_forest_plot from the two-sample MR package [31]. The plots can be found in the Supplementary Data, Figure S1 (vitamin D levels) and Figure S2 (vitamin D deficiency).

### 2.5. Vitamin D Metabolic Pathway Variants

Since vitamin D exists as different metabolites in the body, we investigated the association between SNPs on the gene loci involved in the metabolic pathway of vitamin D and POAG. The genes we investigated were *CYP2R1*, *CYP24A1* and *CYP27B1*, which play sequential roles in converting vitamin D3 into its active hormonal form, calcitriol [36]. We used the National Library of Medicine Genome Browser (https://www.ncbi.nlm.nih.gov/genome/, accessed on 10 October 2023) to find the coordinates of the genes at Genome Reference Consortium Human Build *37* and added 5 kilobases to either end. For *CYP2R1*, the range of position was between chromosome 11:14,903,986–14,918,989, whereas the ranges for *CYP27A1* and *CYP27B1* were chromosome 2:219,651,870–219,685,016, and chromosome 12:58,161,117 to 58,165,861, respectively. We searched for the SNPs within the respective range in the POAG GWAS results referring to Gharahkhani et al. [29]. To correctly identify the number of independent tests performed for each outcome, we clumped the SNPs for each gene with an R^2^ > 0.001. In the case of *CYP2R1*, *CYP27A1*, and *CYP27B1* for the POAG, after clumping, we were left with 62, 6, and 37 independent SNPs, respectively (Supplementary Table S7A–C). Accordingly, the adjusted *p*-value threshold of association used was 4.76 × 10^−4^.

### 2.6. Sensitivity Analysis

#### 2.6.1. Replication Cohort: FinnGen R9

We performed a number of sensitivity tests. Firstly, we obtained POAG summary statistics for our identified instruments from the FinnGen Data Release 9 (R9) as a replication cohort and performed MR against vitamin D levels and deficiency as described above (Supplementary Table S5).

FinnGen is a partnership that combines genome data from a variety of Finnish biobanks and health registries in Finland [37,38]. In total, there were 377,277 individuals. The sample sizes regarding POAG included 7756 cases and 358,375 controls [37,38].

The clinical endpoints for the outcome, as stated in https://www.finngen.fi/en/researchers/clinical-endpoints (accessed on 27 June 2024), are in accordance with the ICD-10 (International Classification of Diseases, Tenth Revision) 2016 version. POAG contains the ICD-10 code H40.1 (POAG) only and excludes other glaucomas (H4[0-2]) (https://icd.who.int/browse10/2016/en#/H40-H42, accessed on 27 June 2024).

#### 2.6.2. Vitamin D Levels *p*-Value Threshold

To test the stability of our estimates, we applied a less stringent *p*-value threshold of 5 × 10^−5^ instead of 5 × 10^−8^ to explore the association between vitamin D level-related SNPs and POAG risk in both Gharahkhani et al. [29] and FinnGen GWAS datasets (Supplementary Tables S4A and S6A, respectively). This *p*-value threshold was not applied to SNPs of vitamin D deficiency due to the lack of full summary statistics available.

#### 2.6.3. Vitamin D Deficiency Winter Samples

To account for seasonal variability, a subset of samples from the winter season was used to re-evaluate the association between the 15 vitamin D deficiency-related genetic variants and POAG [27] (Supplementary Tables S4B and S6B testing for Gharahkhani et al. [29] and FinnGen [37] GWASs, respectively). The effect sizes from this analysis were employed to assess the robustness of findings with respect to seasonality against both datasets.

## 3. Results

### 3.1. Levels of Vitamin D and POAG

We extracted 554 genetic variants from the SUNLIGHT consortium [25] associated with vitamin D levels with a *p*-value threshold of 5 × 10^−8^. These represented eight independent loci with a linkage disequilibrium of less than 0.001 between them. After performing harmonisation with the POAG data, seven independent SNPs remained (Supplementary Table S1A). According to a previous study by Amin & Drenos that used 6 of the 7 SNPs, these instruments are expected to explain more than 2.52% of the variance in vitamin D levels [27]. No evidence of association with potential confounders was observed.

**Figure 1:**
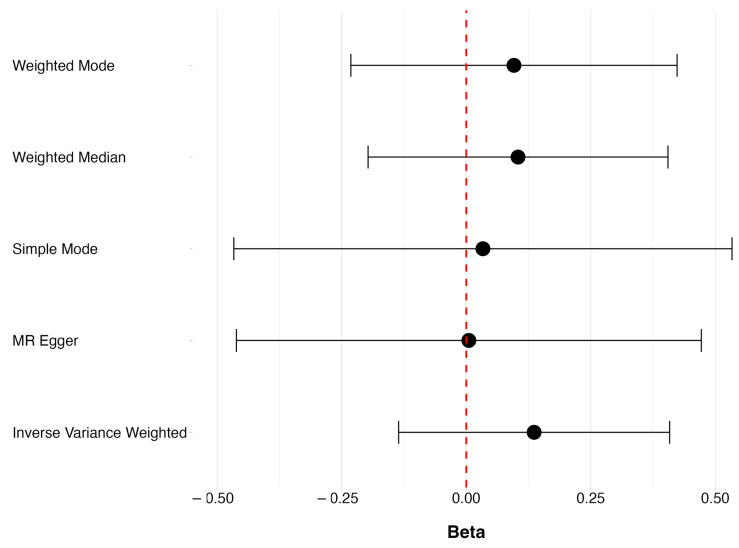
The overall causal effects of vitamin D levels on POAG, showing beta (ln(OR)) and respective 95% CIs for the robust MR methods.

There was no evidence to suggest that increasing vitamin D decreased the risk of POAG (OR = 1.146, 95% CI 0.873 to 1.504, *p* = 0.326). Robust methods of MR, such as MR Egger, the weighted median, the weighted mode, and the simple mode, also found no evidence of a causal relationship between vitamin D levels, as seen in Figure 1. Testing for the violation of our MR assumptions due to pleiotropy and using the intercept of the MR Egger model suggested no evidence of pleiotropy affecting our estimates (*p*-value: 0.528) (Supplementary Table S3A). The causal estimate of each individual SNP for vitamin D levels on POAG can be found in Supplementary Figure S1.

Using a less stringent *p*-value threshold of 5 × 10^−5^, 1255 genetic variants were extracted from the SUNLIGHT consortium [25], of which 84 were independent loci. Following harmonisation, 71 SNPs remained. We again found no evidence of a causal association between vitamin D levels and POAG under any of the MR methods (OR = 1.301, 95% CI 0.951 to 1.777, *p* = 0.099) (Supplementary Table S4A).

### 3.2. Vitamin D Deficiency and POAG

It has been suggested that it is the deficiency of vitamin D, rather than the overall levels of it, that damages eye health [16,17,18,19,20]. By using independent variants of vitamin D deficiency from the UKBB identified by Amin & Drenos [27], we performed a two-sample MR analysis to determine if there was a causal effect of vitamin D deficiency. The authors reported that the SNPs showed association at the 5 × 10^−8^ *p*-value threshold and were independent of each other [27]. These instruments explained 2.11% of the variance in vitamin D deficiency [27]. Based on this information, the SNPs were not clumped further, nor was a lower *p*-value threshold applied for this list of SNPs. After harmonisation between vitamin D deficiency with the POAG data, 15 SNPs remained, which were used as genetic instruments in a two-sample MR (Supplementary Table S1B). No evidence of association with potential confounders was observed.

**Figure 2:**
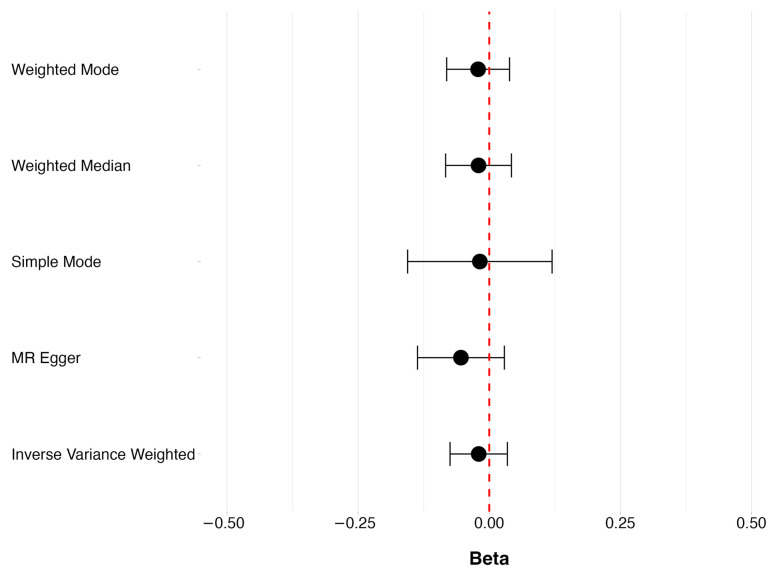
The overall causal effect of vitamin D deficiency POAG, showing beta (ln(OR)) and respective 95% CIs for the robust MR methods.

No evidence was found to suggest a causal relationship between vitamin D deficiency and POAG, as seen in Figure 2, where OR = 0.980 (95% CI 0.928 to 1.036, *p* = 0.471). None of the pleiotropy robust MR methods showed evidence of a causal relationship between vitamin D deficiency and POAG. Using the MR Egger intercept to test for the presence of pleiotropy suggested that our estimates were not biased due to the violation of the pleiotropy assumption (*p*-value 0.307) (Supplementary Table S3B). The causal estimate of each individual SNP of vitamin D deficiency on POAG can be found in Supplementary Figure S2.

### 3.3. Sensitivity Analyses

Using the effect sizes of the 15 SNPs from winter samples only did not alter our results for vitamin D deficiency on POAG risk (OR = 0.982, 95% CI 0.936 to 1.030, *p* = 0.47) (Supplementary Table S4B).

For further sensitivity analyses, we explored the FinnGen R9 GWAS dataset [37] and found no evidence of a causal association between vitamin D levels (OR = 1.192, 95% CI 0.831 to 1.709, *p* = 0.34) and vitamin D deficiency (OR = 0.958, 95% CI 0.879 to 1.045, *p* = 0.33) with POAG risk under any of the five MR methods (Supplementary Tables S5A and S5B, respectively). After implementing a less stringent *p*-value threshold for vitamin D levels, only the weighted median method showed evidence of statistically significant increasing risk with higher vitamin D (OR = 1.587, 95% CI 1.040 to 2.423, *p* = 0.03) (Supplementary Table S6A). Though, there is no evidence that our main IVW estimate (OR = 1.301, 95% CI 0.951 to 1.777, *p* = 0.10) was affected by pleiotropy to accept the weighted median estimate as our most appropriate result. Only considering the effect sizes of winter samples in the case of vitamin D deficiency resulted in no association with POAG under any of the MR methods (IVW OR = 0.963, 95% CI 0.892 to 1.039, *p* = 0.33 and Supplementary Table S6B).

As vitamin D is present in the body in a number of different metabolites not fully represented by the presently used measure, we searched for evidence of an association between SNPs in genes involved in its metabolic pathway and glaucoma [39]. We looked up 62, 6 and 37 independent SNPs in and around *CYP2R1*, *CYP27A1*, and *CYP27B1*, respectively, using Gharahkhani et al.’s [29] GWAS for POAG (Supplementary Table S7). We used multiple test-corrected *p*-value thresholds of 4.76 × 10^−4^ to control for the 105 independent tests. Among these, only one SNP, rs62493714, within *CYP2R1*, met the threshold. We cross-referenced this SNP and other SNPs exhibiting linkage disequilibrium of R^2^ = 1 (https://ldlink.nih.gov/?tab=ldproxy, accessed on 27 June 2024) within the FinnGen data. None of the identified SNPs, including the proxy SNP, exhibited evidence of statistical significance with POAG (Supplementary Table S8). 

## 4. Discussion

Using data from previously published large-scale GWASs for vitamin D levels [25] and vitamin D deficiency [27], we tested the causal effect of these exposures on the risk of POAG diagnosis in individuals of European ancestry. Our results did not provide evidence for the suggested protective functions of vitamin D for the onset of POAG. Even the use of a less stringent *p*-value threshold for genetic variants of vitamin D levels and the use of only effect sizes of winter samples in the case of vitamin D deficiency failed to yield evidence of an association. For the additional sensitivity analysis, we expanded our analysis to the FinnGen R9 GWAS dataset [37]. We again did not observe any evidence of a protective association. Overall, our results suggest that is unlikely that increased vitamin D levels can protect from glaucoma.

Further, we examined specific genetic markers in three genes related to vitamin D processing (*CYP2R1*, *CYP27A1*, and *CYP27B1*). Only one SNP (rs62493714) in the *CYP2R1* gene showed a possible link to POAG, but this did not replicate in the FinnGen GWAS [37]. Hence, we found no evidence of a robust association between genetic variants in genes responsible for the key steps of vitamin D metabolism, suggesting that other vitamin D metabolites, beyond the measured 25(OH)D, are also unlikely to modify POAG risk. Contrary to the hypothesised associations, the results of our study suggest that increasing vitamin D concentrations are unlikely to lead to a protective effect against POAG.

Although previous studies [16,17,18,19,20] have suggested an association between serum vitamin D levels and vitamin D deficiency with glaucoma, in most cases, the approach used cannot distinguish between correlation and causation or assess vitamin D as an independent risk factor of either glaucoma incidence, progression, or severity. In a recent case–control study on participants of African ancestry, it was found that those with advanced glaucoma had lower free 25(OH)D levels than those with early glaucoma and normal subjects [16]. In another case–control study performed on European ancestry patients, an association between the presence, but not the severity of the disease, was found between POAG and 25(OH)D levels, where glaucoma patients had 15% lower serum 25(OH)D levels in comparison to the healthy controls [18]. Despite the presence of this correlation, it is unclear if the results can be interpreted as vitamin D protecting people from glaucoma or whether glaucoma patients prefer to remain indoors, especially if they suffer from visual impairment, or if a third unobserved variable is responsible for the observed result. Such observational studies often struggle with confounding factors and reverse causation, which our MR study aimed to address by testing causality more robustly. Therefore, the discrepancies between our findings and earlier studies could be attributed to the differences in study design, methodology, and population characteristics. A randomised control trial [40], which tested the supplementation of cholecalciferol (40,000 IU) for a period of six months as a means to lower the IOP level—and thus glaucoma progression— showed no significant difference in IOP levels. The results of this study suggest that higher doses of vitamin D have no effect on IOP levels and, to an extent, glaucoma progression, thus confirming our observed results that there is no protective effect of vitamin D [40]. The agreement between randomised control trials and MR studies is to be expected, as MR was previously described as nature’s randomised control study, and the correspondence between the two has been shown in several other cases [22,23,24].

It has been previously suggested that levels of 25(OH)D are not representative of the true effect of vitamin D on our health [36]. Here, we also used variants in the CYP2R1, *CYP27A1* and *CYP27B1* genes for evidence of association with glaucoma. These three genes give a better indication and insight into the metabolism of vitamin D. *CYP2R1* and *CYP27A1* code for enzymes involved in the first hydroxylation to produce 25(OH)D, whilst *CY27B1* codes for the enzyme that further hydroxylates 25(OH)D into the active form of vitamin D, 1,25(OH)2 [6]. The results of our investigation confirmed our MR conclusions of no detectable effect on the used data.

A number of limitations are present. Foremost, our findings cannot be applied to the relationship between vitamin D levels or deficiency with glaucoma risk in non-European ancestry groups. The exposure instruments (vitamin D levels and vitamin D deficiency) and the outcome data were extracted from GWASs of individuals of European ancestry [25,27]. Because the exposure data were composed of European ancestry subjects, this meant that the outcome datasets also had to be from those of a European ancestry. This ancestry-specific limitation means that our results may not be applicable to populations of non-European descent. Due to genetic and environmental variation, such as those related to skin pigmentation and UVB exposure, important differences may exist. To address this limitation, expanding research through international collaborations and large-scale multi-ethnic GWASs could provide more robust and generalizable insights. Future MR studies could repeat this analysis on other ancestry groups to validate whether observed associations hold across different ancestral backgrounds. The focus should be on those of African ancestry, as this ancestry is considered a risk factor for glaucoma [41]. It would be interesting to examine if this risk is mediated by lower vitamin D levels since those with darker skin require more UVB exposure to absorb vitamin D [36].

In conclusion, although vitamin D is considered an etiological factor in models of inflammation, immunomodulation, and neurotransmission pathways, we found no evidence to suggest the causal role of vitamin D levels or its deficiency on POAG. Previously, it was hoped that vitamin D supplementation could be used as a relatively safe and affordable preventive intervention for glaucoma. However, as also suggested by smaller randomised control trials [40] and confirmed here using much larger population samples, this is not supported by the current evidence. Clinically, this suggests that practitioners should not rely on vitamin D as a preventive measure and should instead focus on other established risk factors and evidence-based treatments for POAG. Our results highlight the need to identify other risk factors with reliable causal evidence that could serve as targets for effective interventions. 

## Data Availability

The original contributions presented in the study are included in the article, further inquiries can be directed to the corresponding author.

## Supplementary Materials

The following supporting information can be downloaded at https://www.mdpi.com/article/10.3390/genes15081084/s1, Figure S1: The MR effect sizes and 95% CI of the causal estimate of each independent SNP of the 7 variants associated with vitamin D concentrations from Jiang et al. on POAG outcome, in comparison to the causal effect as estimated using the MR Egger and IVW (outcome: PMID 33627673 GCST90011767 summary statistics); Figure S2: The MR effect sizes and 95% CI of the causal estimate of each independent SNP of the 15 variants associated with vitamin D deficiency from Amin & Drenos on POAG outcome, in comparison to the causal effect as estimated using the MR Egger and IVW (outcome: PMID 33627673 GCST90011767 summary statistics); Table S1: Regression coefficients (Beta), or log odds ratios, and standard errors (SE) when regressing the number of effect alleles carried (or their dosage for impute genetic variants) at a single nucleotide polymorphism genomic position on natural-log transformed levels of 25-hydroxyvitamin D or vitamin D deficiencY; Table S2: Regression coefficients (Beta), or log odds ratios, and standard errors (SE) when regressing the number of effect alleles carried (or their dosage) at a single nucleotide polymorphism genomic position on natural-log transformed levels of 25-hydroxyvitamin D or vitamin D deficiency; Table S3: Log odds ratio (Beta), *p*-values (Pval), standard error (SE), lower (Lower) and upper (Upper) 95% confidence intervals and MR Egger intercept *p*-values (P_intercept) from a two-sample MR analysis of the effect of vitamin D levels and vitamin D deficiency on POAG (outcome) using PMID 33627673 GCST90011767 summary statistics; Table S4: Log odds ratio (Beta), *p*-values (Pval), standard error (SE), lower (Lower) and upper (Upper) 95% confidence intervals and MR Egger intercept *p*-values (P_intercept) from a two-sample MR analysis of the effect of vitamin D levels (at *p* value threshold 5 × 10^−5^) and vitamin D deficiency (using winter samples only) on POAG using PMID 33627673 GCST90011767 summary statistics; Table S5: Log odds ratio (Beta), *p*-values (Pval), standard error (SE), lower (Lower) and upper (Upper) 95% confidence intervals and MR Egger intercept *p*-values (P_intercept) from a two-sample MR analysis of the effect of vitamin D levels and vitamin D deficiency on POAG using FinnGen R9 POAG summary statistics; Table S6: Log odds ratio (Beta), *p*-values (Pval), standard error (SE), lower (Lower) and upper (Upper) 95% confidence intervals and MR Egger intercept *p*-values (P_intercept) from a two-sample MR analysis of the effect of vitamin D levels (at *p* value threshold 5 × 10^−5^) and vitamin D deficiency (using winter samples only) on POAG using FinnGen R9 POAG summary statistics; Table S7: Independent SNPs (SNP) of *CYP2R1*, *CYP27A1* and *CYP27B1*, and their positions (SNP_Position) and respective *p*-values (Pval) in the PMID 33627673 GCST90011767 summary statistics; Table S8: SNP, effect allele (Effect_Allele), other allele (Other_Allele), log odds ratio (Beta), standard error (SE), and *p*-values (Pval) results of independent SNP of *CYP2R1* that was significant from Table S7A (rs62493714) in PMID 33627673 GCST90011767 summary statistics and after being cross-referenced in the FinnGen data, as well as results of another SNP exhibiting a linkage disequilibrium of R^2^ = 1.
