# Supplementary material for: No Evidence That Vitamin D Levels or Deficiency Are Associated with the Risk of Open-Angle Glaucoma in Individuals of European Ancestry: A Mendelian Randomisation Analysis
Source: Genes (Basel). 2024 Aug 16;15(8):1084. doi: 10.3390/genes15081084 (PMC11354120; doi:10.3390/genes15081084)

SUPPLEMENTARY MATERIAL

Supplementary Table S1

Regression coefficients (Beta), or log odds ratios, and standard errors (SE) when regressing the number of effect alleles carried (or their dosage for impute genetic variants) at a single nucleotide polymorphism genomic position on natural-log transformed levels of 25-hydroxyvitamin D or vitamin D deficiency respectively. The use of the effect alleles as predictors is called the additive model and is the one predominately, if not exclusively, used in genome wide association studies of common complex diseases and associated risk factors. Each SNP is tested separately and adjusted by study specific covariates, commonly age, sex and principle components describing the structure of the genome wide data capturing the genetic stratification of the genotyped sample:

(A) vitamin D concentrations on the 7 independent variants associated with vitamin D concentrations from Jiang *et al*. Note that these SNPs are at p-value threshold of 5×10^{-8} and have been clumped at $R^2 > 0.001$. The model included month of sample collection, age, sex, body mass index, and principal components capturing genetic ancestry as covariates.

(B) vitamin D deficiency on the 15 independent variants associated with vitamin D deficiency in the UK Biobank from Amin & Drenos. The original paper had 17 SNPs, but rs3755322 and rs964184 were removed for being palindromic with intermediate allele frequencies at the harmonisation step. Note that these SNPs are at p-value threshold of 5×10^{-8} and have been clumped at $R^2 > 0.001$ in the source publication. The logistic model included the first four principal components for the genetic variability of the genome, age at baseline, sex and the genotyping array used as covariates.

(A)

SNP	Effect Allele	Beta	SE	Pval	Other Allele
rs10741657	A	0.0308	0.0022	2.05E-46	G
rs10745742	T	0.0165	0.0022	1.88E-14	C
rs12785878	T	0.0363	0.0022	3.81E-62	G
rs17216707	T	0.0263	0.0027	8.14E-23	C
rs17467825	A	0.0902	0.0024	1.00E-200	G
rs2597193	A	0.0187	0.0022	6.26E-17	G
rs6839473	A	0.0174	0.0022	1.79E-15	G

(B)

SNP	Effect Allele	Beta	SE	Pval	Other Allele
rs7129781	C	0.189	0.016	3.53E-33	T
rs4944958	A	0.259	0.01	8.74E-143	G
rs10859995	T	-0.105	0.009	3.01E-33	C
rs1532085	A	0.05	0.009	1.15E-08	G
rs1800588	T	0.066	0.01	1.92E-10	C
rs55791371	C	-0.081	0.013	1.27E-09	A
rs10426201	G	-0.106	0.012	8.17E-20	A
rs3750297	A	0.059	0.009	2.72E-10	G
rs12123821	T	-0.227	0.022	6.25E-26	C
rs4845491	C	-0.119	0.019	7.46E-10	T
rs8123293	G	-0.076	0.014	2.52E-08	A

rs17217119	G	0.086	0.011	4.95E-16	A
rs6600893	C	0.067	0.009	5.49E-15	T
rs2282679	G	0.429	0.009	1.00E-200	T
rs2205262	C	-0.057	0.009	5.13E-11	A

Supplementary Table S2

Regression coefficients (Beta), or log odds ratios, and standard errors (SE) when regressing the number of effect alleles carried (or their dosage) at a single nucleotide polymorphism genomic position on natural-log transformed levels of 25-hydroxyvitamin D or vitamin D deficiency respectively:

(A) vitamin D concentrations on the 71 independent variants associated with vitamin D concentrations from Jiang *et al*. Note that these SNPs are at p-value threshold of 5 x 10^{-5} and have been clumped at R^2 > 0.001. The model included month of sample collection, age, sex, body mass index, and principal components capturing genetic ancestry as covariates. **(B)** vitamin D deficiency on the 15 independent variants associated with vitamin D deficiency in the UK Biobank from Amin & Drenos using winter samples only. The original paper had 17 SNPs, but 2 (rs3755322 and rs964184) were removed for being palindromic with intermediate allele frequencies at the harmonisation step. Note that these SNPs are at p-value threshold of 5 x 10^{-8} and have been clumped at R^2 > 0.001. The logistic model included the first four principal components for the genetic variability of the genome, age at baseline, sex and the genotyping array used as covariates

(A)

SNP	Effect Allele	Beta	SE	Pval	Other Allele
rs6982502	T	-0.0094	0.002	4.04E-06	C
rs7038954	T	-0.0145	0.0033	1.50E-05	C
rs12571703	A	-0.0112	0.0027	4.55E-05	G
rs6678054	A	-0.0315	0.0075	2.64E-05	C
rs12785878	T	0.0363	0.0022	3.81E-62	G
rs7670348	T	-0.0118	0.0029	3.78E-05	C
rs7781168	A	0.0096	0.002	2.95E-06	G
rs3856858	T	-0.0088	0.0022	4.55E-05	G
rs7831091	T	0.0201	0.0046	1.09E-05	G
rs11981000	A	0.0082	0.002	4.82E-05	G
rs9589953	A	0.0198	0.0048	3.08E-05	G
rs12561867	A	-0.0191	0.0046	3.34E-05	G
rs8083994	T	-0.0091	0.0021	1.57E-05	G
rs617172	A	-0.0122	0.0028	1.11E-05	G
rs11690961	A	0.013	0.0032	4.94E-05	C
rs1475447	T	0.0106	0.0026	3.50E-05	C
rs2223200	A	-0.0105	0.0025	2.55E-05	G
rs904856	A	0.0228	0.0045	4.12E-07	G
rs914787	T	0.0105	0.0022	1.25E-06	C
rs10890384	T	-0.0127	0.0029	1.16E-05	C
rs828929	A	-0.0088	0.0022	4.97E-05	G
rs6768085	T	-0.0104	0.0025	4.03E-05	C
rs2839288	A	0.0098	0.0024	3.60E-05	G
rs1876504	A	-0.0711	0.0173	3.94E-05	G
rs2890689	A	0.0108	0.0026	4.19E-05	G

rs2597193	A	0.0187	0.0022	6.26E-17	G
rs3735789	A	-0.0106	0.0024	1.20E-05	C
rs11203339	T	-0.0104	0.0021	8.56E-07	C
rs793000	A	0.0099	0.0022	4.90E-06	G
rs6770113	A	0.0098	0.0023	1.77E-05	C
rs6780224	T	-0.0105	0.0023	4.46E-06	C
rs17023019	A	0.009	0.0021	1.89E-05	G
rs9358923	A	-0.0111	0.0026	2.56E-05	G
rs12572275	T	-0.0302	0.0069	1.01E-05	C
rs7027321	T	0.0089	0.0022	4.49E-05	G
rs2421345	A	-0.0086	0.0021	4.10E-05	C
rs4401964	T	-0.0096	0.0022	1.00E-05	C
rs17101099	A	-0.0211	0.005	2.34E-05	G
rs10888491	A	0.012	0.0022	8.62E-08	G
rs10745742	T	0.0165	0.0022	1.88E-14	C
rs16897644	A	0.0119	0.0028	2.23E-05	G
rs7653527	A	-0.0151	0.0036	2.44E-05	G
rs17159572	A	0.1242	0.028	9.09E-06	G
rs9881542	A	0.0104	0.0025	3.67E-05	C
rs4293393	A	0.0111	0.0025	1.13E-05	G
rs11963334	T	-0.0176	0.0042	2.57E-05	G
rs1892419	T	-0.0104	0.0025	2.54E-05	C
rs3783321	T	0.0086	0.0021	2.94E-05	G
rs10741657	A	0.0308	0.0022	2.05E-46	G
rs17084391	T	-0.0092	0.0022	4.19E-05	C
rs6839473	A	0.0174	0.0022	1.79E-15	G
rs11174335	T	0.0096	0.0023	3.98E-05	G
rs2303918	A	0.0082	0.002	3.38E-05	G
rs17568679	T	-0.0124	0.0029	1.44E-05	C
rs1736924	T	0.0155	0.0038	4.53E-05	C
rs465096	A	0.0087	0.0021	4.27E-05	G
rs12768603	A	-0.0128	0.0031	3.52E-05	G
rs2972619	T	0.013	0.0029	6.85E-06	C
rs6892909	A	-0.0207	0.0048	1.93E-05	G
rs6657893	A	0.0097	0.0023	1.87E-05	G
rs11654241	T	0.0112	0.0025	5.89E-06	C
rs724675	A	-0.0107	0.0026	4.83E-05	C
rs17216707	T	0.0263	0.0027	8.14E-23	C
rs2914087	T	-0.0142	0.0032	1.15E-05	C

rs17467825	A	0.0902	0.0024	1.00E-200	G
rs697485	A	-0.0118	0.0028	2.65E-05	C
rs999888	A	-0.0102	0.0024	1.95E-05	G
rs1322987	T	0.0088	0.0021	2.67E-05	C
rs6752251	T	0.0088	0.0022	4.71E-05	G
rs4821976	A	0.01	0.0021	2.74E-06	G
rs11872638	T	-0.0109	0.0027	4.23E-05	G

(B)

SNP	Effect Allele	Beta	SE	Pval	Other Allele
rs7129781	C	0.072	0.159	0.032	6.01E-07
rs4944958	A	0.208	0.283	0.02	5.91E-44
rs10859995	T	0.413	-0.116	0.017	7.47E-12
rs1532085	A	0.384	0.043	0.017	1.20E-02
rs1800588	T	0.215	0.086	0.02	1.81E-05
rs55791371	C	0.119	-0.092	0.026	3.51E-04
rs10426201	G	0.17	-0.132	0.022	3.17E-09
rs3750297	A	0.288	0.069	0.018	1.62E-04
rs12123821	T	0.046	-0.256	0.041	4.88E-10
rs4845491	C	0.055	-0.12	0.037	1.23E-03
rs8123293	G	0.113	-0.092	0.027	5.41E-04
rs17217119	G	0.194	0.098	0.021	2.76E-06
rs6600893	C	0.456	0.073	0.017	1.24E-05
rs2282679	G	0.284	0.504	0.019	6.81E-162
rs2205262	C	0.428	-0.065	0.017	1.03E-04

Supplementary Table S3

Several MR methods are employed to estimate a causal effect. Each method provides an estimate of the log odds ratio (Beta), which reflects the association between the predictors and the outcomes.

Log odds ratio (Beta), p-values (Pval), standard error (SE), lower (Lower) and upper (Upper) 95% confidence intervals and MR Egger intercept p-values (P_intercept) from a two-sample MR analysis of the effect of

(A) vitamin D levels (predictor)

(B) vitamin D deficiency (predictor)

on POAG (outcome) using PMID 33627673 GCST90011767 summary statistics.

A.

Outcome	Method	Beta	SE	Lower	Upper	Pval	P_Intercept
POAG	MR Egger	0.005	0.238	-0.461	0.472	0.983	0.528
POAG	Weighted Median	0.104	0.152	-0.195	0.403	0.495	N/A
POAG	Inverse Variance Weighted	0.136	0.139	-0.136	0.408	0.326	N/A
POAG	Simple Mode	0.033	0.243	-0.443	0.510	0.895	N/A
POAG	Weighted Mode	0.096	0.156	-0.209	0.401	0.561	N/A

B.

Outcome	Method	Beta	SE	Lower	Upper	Pval	P_Intercept
POAG	MR Egger	-0.054	0.042	-0.137	0.029	0.223	0.307
POAG	Weighted Median	-0.020	0.032	-0.083	0.042	0.519	N/A
POAG	Inverse Variance Weighted	-0.020	0.028	-0.075	0.035	0.471	N/A
POAG	Simple Mode	-0.018	0.068	-0.152	0.116	0.797	N/A
POAG	Weighted Mode	-0.021	0.030	-0.079	0.037	0.484	N/A

Supplementary Table S4

Log odds ratio (Beta), p-values (Pval), standard error (SE), lower (Lower) and upper (Upper) 95% confidence intervals and MR Egger intercept p-values (P_intercept) from a two-sample MR analysis of the effect of

(A) vitamin D levels (at p value threshold 5 x 10^{-5})

(B) vitamin D deficiency (using winter samples only)

on POAG using PMID 33627673 GCST90011767 summary statistics.

A.

Outcome	Method	Beta	SE	Lower	Upper	Pval	P_Intercept
POAG	MR Egger	0.165	0.203	-0.232	0.562	0.418	0.381
POAG	Weighted Median	0.112	0.151	-0.184	0.409	0.458	N/A
POAG	Inverse Variance Weighted	0.030	0.132	-0.230	0.290	0.821	N/A
POAG	Simple Mode	-0.162	0.390	-0.927	0.604	0.680	N/A
POAG	Weighted Mode	0.071	0.146	-0.216	0.358	0.630	N/A

B.

Outcome	Method	Beta	SE	Lower	Upper	Pval	P_Intercept
POAG	MR Egger	-0.046	0.036	-0.117	0.026	0.233	0.321
POAG	Weighted Median	-0.020	0.028	-0.075	0.035	0.484	N/A
POAG	Inverse Variance Weighted	-0.018	0.024	-0.066	0.030	0.470	N/A
POAG	Simple Mode	-0.013	0.065	-0.141	0.115	0.844	N/A
POAG	Weighted Mode	-0.019	0.026	-0.069	0.031	0.465	N/A

Supplementary Table S5

Log odds ratio (Beta), p-values (Pval), standard error (SE), lower (Lower) and upper (Upper) 95% confidence intervals and MR Egger intercept p-values (P_intercept) from a two-sample MR analysis of the effect of

(A) vitamin D levels

(B) vitamin D deficiency

on POAG using FinnGen R9 POAG summary statistics.

A.

Outcome	Method	Beta	SE	Lower	Upper	Pval	P_Intercept
POAG	MR Egger	0.506	0.315	-0.112	1.124	0.169	0.260
POAG	Weighted Median	0.392	0.223	-0.045	0.828	0.079	N/A
POAG	Inverse Variance Weighted	0.176	0.184	-0.185	0.536	0.340	N/A
POAG	Simple Mode	0.363	0.488	-0.594	1.320	0.485	N/A
POAG	Weighted Mode	0.453	0.243	-0.024	0.930	0.112	N/A

B.

Outcome	Method	Beta	SE	Lower	Upper	Pval	P_Intercept
POAG	MR Egger	-0.072	0.070	-0.209	0.065	0.320	0.589
POAG	Weighted Median	-0.077	0.047	-0.168	0.014	0.098	N/A
POAG	Inverse Variance Weighted	-0.043	0.044	-0.129	0.044	0.333	N/A
POAG	Simple Mode	-0.018	0.097	-0.208	0.171	0.851	N/A
POAG	Weighted Mode	-0.066	0.043	-0.150	0.019	0.150	N/A

Supplementary Table S6

Log odds ratio (Beta), p-values (Pval), standard error (SE), lower (Lower) and upper (Upper) 95% confidence intervals and MR Egger intercept p-values (P_intercept) from a two-sample MR analysis of the effect of

(A) vitamin D levels (at p value threshold 5 x 10^{-5})

(B) vitamin D deficiency (using winter samples only)

on POAG using FinnGen R9 POAG summary statistics.

A.

Outcome	Method	Beta	SE	Lower	Upper	Pval	P_Intercept
POAG	MR Egger	0.278	0.259	-0.229	0.785	0.286	0.940
POAG	Weighted Median	0.462	0.216	0.039	0.885	0.032	N/A
POAG	Inverse Variance Weighted	0.263	0.159	-0.050	0.575	0.099	N/A
POAG	Simple Mode	0.511	0.506	-0.480	1.503	0.316	N/A
POAG	Weighted Mode	0.359	0.205	-0.042	0.760	0.083	N/A

B.

Outcome	Method	Beta	SE	Lower	Upper	Pval	P_Intercept
POAG	MR Egger	-0.063	0.060	-0.181	0.055	0.316	0.592
POAG	Weighted Median	-0.074	0.040	-0.153	0.005	0.067	N/A
POAG	Inverse Variance Weighted	-0.038	0.039	-0.114	0.038	0.326	N/A
POAG	Simple Mode	-0.016	0.091	-0.195	0.163	0.862	N/A
POAG	Weighted Mode	-0.063	0.040	-0.142	0.017	0.145	N/A

Supplementary Table S7

(A) Independent SNPs (SNP) of *CYP2R1* and their positions (SNP_Position) and respective p-values (Pval) in the PMID 33627673 GCST90011767 summary statistics.

SNP	SNP Position	Pval
rs80131117	14906696	0.4106
rs116100114	14906840	0.1913
rs143030913	14909418	0.4097
rs115923905	14912974	0.04337
rs12740958	14914022	0.5478
rs151228099	14917216	0.09572
rs34139468	14906579	0.8132
rs139461614	14906663	0.3552
rs116542673	14909505	0.002964
rs148961165	14912434	0.5044
rs116603004	14914481	0.4725
rs145554539	14909364	0.01327
rs147631414	14909899	0.2023
rs293917	14918423	0.003199
rs62410830	14905644	0.02326
rs77995215	14909508	0.2575
rs9312875	14909219	0.00313
rs76979760	14906756	0.3231
rs62390352	14908323	0.4651
rs74799037	14909353	0.02395
rs75340873	14911544	0.619
rs217570	14917724	0.005773
rs62493714	14907800	0.0002222
rs10108640	14913465	0.01528
rs1412725	14906086	0.04711
rs117939007	14908640	0.2613
rs115626683	14910618	0.2288
rs72772424	14910732	0.1161
rs11259371	14914673	0.3205
rs76593453	14918458	0.1325
rs1562902	14918216	0.1488
rs138326953	14904096	0.2703
rs61920237	14904432	0.02294
rs117789323	14914634	0.4907
rs150371334	14917997	0.1221
rs140683400	14906267	0.4358
rs568505519	14912438	0.8491
rs369983233	14916055	0.4909
rs111291085	14916780	0.233
rs183383157	14917177	0.268
rs150496208	14909021	0.03084
rs17714088	14909549	0.2423
rs8075571	14909688	0.4711
rs76407643	14910043	0.3492
rs117373117	14910570	0.2258

rs75381851	14911445	0.4303
rs74804082	14913797	0.8273
rs62061379	14913808	0.5093
rs147708384	14918425	0.8364
rs150757793	14908501	0.3618
rs77310291	14910626	0.5256
rs35349582	14912796	0.0005977
rs144246303	14914786	0.4576
rs111933576	14917771	0.2996
rs9305050	14907953	0.01554
rs117042785	14907623	0.674
rs191480765	14910764	0.4515
rs6135314	14911003	0.0626
rs35910325	14915921	0.5901
rs61701804	14904393	0.07272
rs138530549	14908342	0.9681
rs145086683	14917807	0.6299

(B) Independent SNPs (SNP) of *CYP27A1* and their positions (SNP_Position) and respective p-values (Pval) in the PMID 33627673 GCST90011767 summary statistics.

SNP	SNP Position	Pval
rs1563352	219652795	0.0004901
rs182460951	219658424	0.394
rs11677711	219669375	0.06714
rs34252444	219672837	0.8633
rs151203082	219680290	0.3932
rs191765452	219684732	0.26

(C) Independent SNPs (SNP) of *CYP27B1* and their positions (SNP_Position) and respective p-values (Pval) in the PMID 33627673 GCST90011767 summary statistics.

SNP	SNP Position	Pval
rs74873756	58162853	0.0459
rs74320918	58163362	0.0388
rs141323187	58161478	0.1794
rs12990929	58164777	0.0512
rs73942239	58165830	0.0338
rs6807500	58163235	0.0676
rs11708047	58165283	0.0302
rs4507427	58163349	0.0199
rs2547915	58164772	0.0138
rs143794839	58163761	0.0622
rs144608843	58162381	0.0449

rs72650878	58164626	0.0729
rs2243440	58165650	0.0138
rs7115601	58161312	0.0599
rs112811173	58162163	0.1433
rs117126516	58163987	0.0375
rs118158773	58164008	0.0318
rs118100988	58164285	0.1368
rs10877013	58165085	0.0144
rs111399599	58163806	0.0902
rs112144828	58164147	0.0488
rs9707520	58161998	0.0203
rs144463771	58163785	0.1441
rs142030905	58164687	0.1105
rs8032850	58165181	0.0598
rs118020072	58161230	0.071
rs112501441	58162403	0.0349
rs1847030	58165805	0.0384
rs117331479	58161699	0.039
rs117416675	58161464	0.091
rs78810023	58161890	0.0429
rs1560398	58163189	0.0339
rs76422013	58161757	0.0386
rs11666171	58163739	0.0782
rs62126247	58165417	0.0369
rs13045071	58162908	0.0157
rs6026979	58163842	0.0497

Supplementary Table S8

SNP, effect allele (Effect_Allele), other allele (Other_Allele), log odds ratio (Beta), standard error (SE), and p-values (Pval) results of

(A) independent SNP of *CYP2R1* that was significant from Table S7A (rs62493714) in PMID 33627673 GCST90011767 summary statistics.

SNP	Effect Allele	Other Allele	Beta	SE	Pval
rs62493714	A	T	-0.0934	0.0253	0.000222

(B) After being cross-referenced in the FinnGen data, as well as results of another SNP exhibiting a linkage disequilibrium of R^2= 1.

SNP	Effect Allele	Other Allele	Beta	SE	Pval
rs62493714	A	T	0.0230845	0.0372608	0.535561
rs73665372	C	T	0.0300564	0.039106	0.442138

Supplementary Figure S1

The MR effect sizes and 95% CI of the causal estimate of each independent SNP of the 7 variants associated with vitamin D concentrations from Jiang *et al*. on POAG outcome, in comparison to the causal effect as estimated using the MR Egger and IVW (outcome: PMID 33627673 GCST90011767 summary statistics)

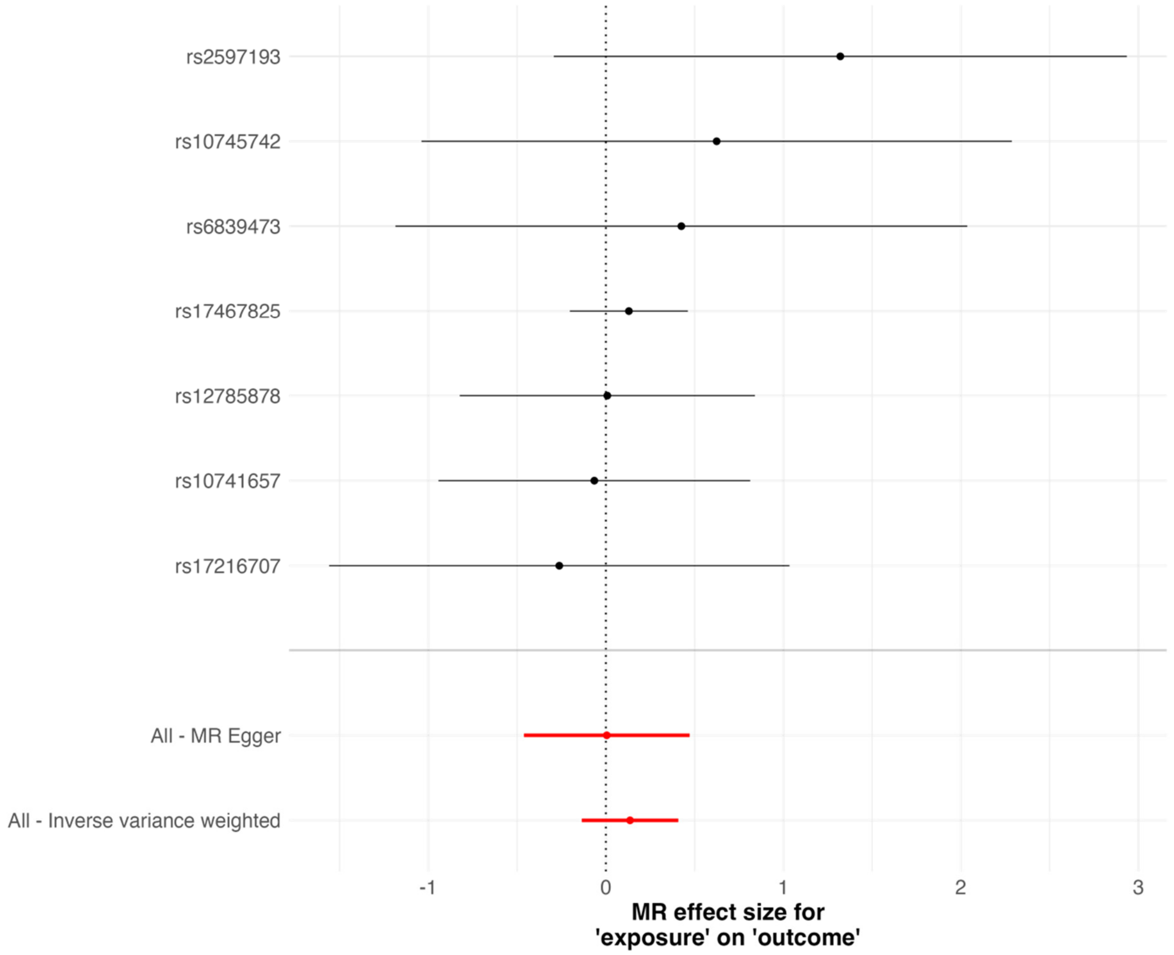

Supplementary Figure S2

The MR effect sizes and 95% CI of the causal estimate of each independent SNP of the 15 variants associated with vitamin D deficiency from Amin & Drenos on POAG outcome, in comparison to the causal effect as estimated using the MR Egger and IVW (outcome: PMID 33627673 GCST90011767 summary statistics)

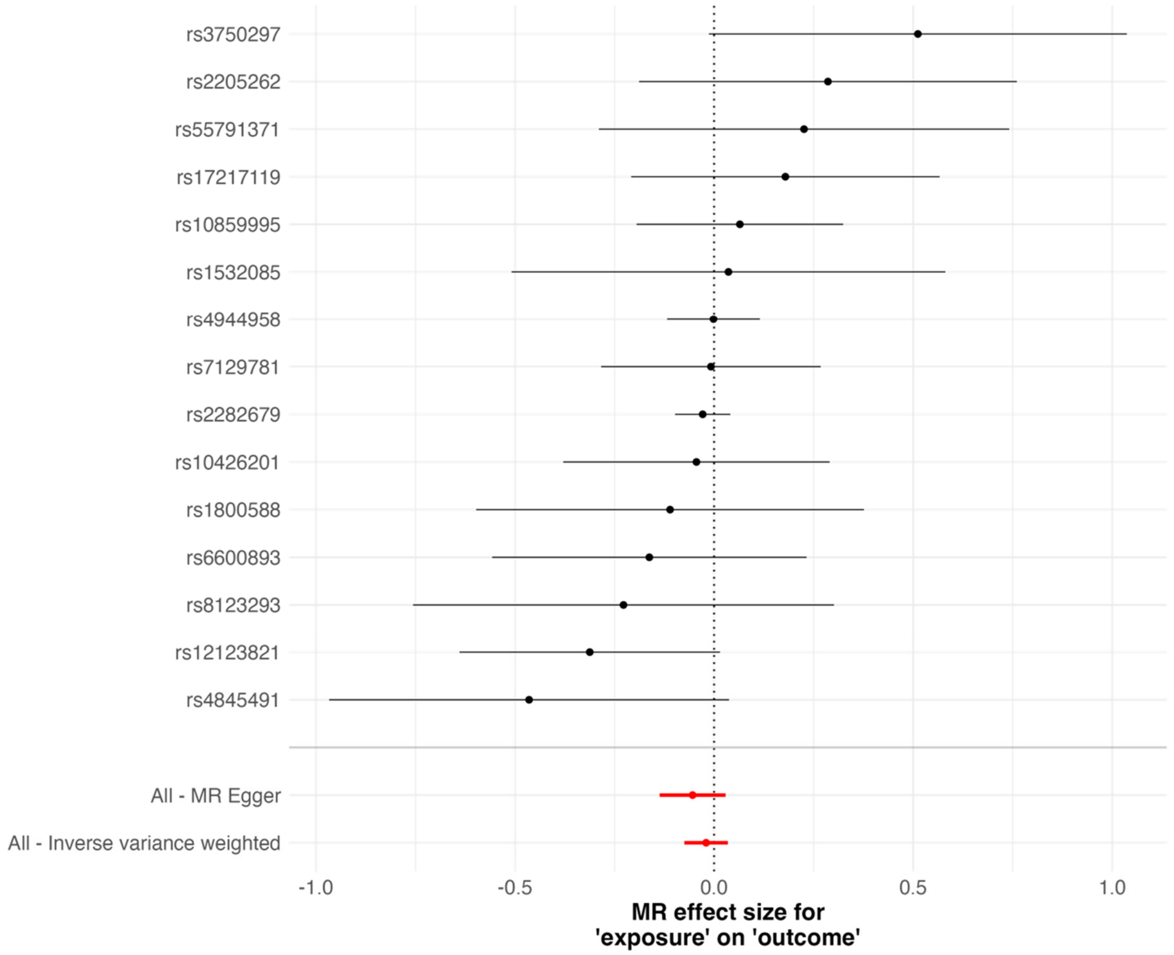

Supplement: Supplementary file 1 [file genes-15-01084-s001.zip › genes-3104997-supplementary.pdf]
